# What Accounts for the Factors of Psychopathology? An Investigation of the Neurocognitive Correlates of Internalising, Externalising, and the *p*-Factor

**DOI:** 10.3390/brainsci12040421

**Published:** 2022-03-22

**Authors:** Darren Haywood, Frank D. Baughman, Barbara A. Mullan, Karen R. Heslop

**Affiliations:** 1 Discipline of Psychology, School of Population Health, Curtin University, Perth 6845, Australia or darren.haywood@svha.org.au (D.H.); frank.baughman@curtin.edu.au (F.D.B.); 2 Mental Health, St. Vincent’s Hospital, Melbourne 3065, Australia; 3 Curtin School of Nursing, Curtin University, Perth 6845, Australia; k.heslop@curtin.edu.au

**Keywords:** *p*-factor, internalising, externalising, psychopathology, neurocognition, executive functioning, working memory, shifting, inhibition, speed of processing

## Abstract

Neurocognitive deficits have been consistently associated with a wide range of psychopathology and are proposed to not only be a consequence of the development of psychopathology but also directly involved in its aetiology. However, there is no clear understanding of what neurocognitive processes are particularly important to mental health. In this paper, we explored the association between neurocognitive abilities and the factors derived from structural models of psychopathology. Four hundred participants from a representative community sample completed measures of symptomology and substance use, as well as 8 neurocognitive tasks. We found a correlated-factors model, with internalising and externalising as the higher-order factors, and a single-factor model with only the *p*-factor, to be good fits for the data. Tasks that measured the speed of processing were significantly associated with internalising, externalising, and the *p*-factor, and accounted for significant amounts of unique variance in the factors after accounting for the common variance of the other tasks. Tasks that measured working memory, shifting, and inhibition were not significantly associated with psychopathology factors. Our findings suggest that neurocognitive abilities may not be differentially associated with psychopathology factors, but that speed of processing is a common correlate of the factors. We emphasise the importance of examining neurocognitive abilities and psychopathology on the individual level.

## 1. Introduction

Neurocognitive abilities refer to cognitive capabilities grounded in particular neurological properties or systems, and include both higher and lower level cognitive processes [1]. Higher level neurocognitive processes include executive functioning that is responsible for the control of mental abilities, including the control of working memory (i.e., *updating*), attention (i.e., *shifting*), and predominant responses (i.e., *inhibition*) [2]. While lower level neurocognitive processes, such as general information processing (i.e., *speed of processing*), are more basic to the system [3]. The proper functioning of neurocognitive abilities, at both higher and lower levels, govern the ability to conduct goal-oriented activity, respond to environmental demands in a timely and appropriate way, and are fundamental to the successful completion of many everyday activities [1]. It is therefore understandable that deficits in neurocognitive performance may result in adverse cognitive and behavioural experiences.

Neurocognitive deficits have been consistently associated with a wide range of psychopathological disorders [4]. Neurocognitive deficits have been proposed to not only be a consequence of the development of psychopathology but also directly involved in the aetiology of psychopathology, e.g., [5,6]. It has been suggested that humans are often exposed to novel and conflicting information. To effectively deal with this information, humans need to stop, reflect, and choose the most appropriate behaviours [6,7,8]. Romer and Pizzagalli [6], Cunningham, Zelazo, Packer, and Van Bavel [7], and Zelazo [8], suggested that the reflection and selection actions require proper neurocognitive performance, including the *updating* of the contents of working memory, *switching* between mental sets, *inhibiting* a predominant response, and effectively *processing information*. Romer and Pizzagalli [6] also proposed that deficits in neurocognitive processes may therefore result in the selection of inappropriate behaviours, poor adaptive ability, and poor conflict resolution, all of which are features of many psychopathologies. Further evidence for neurocognitive abilities being an aeitological feature of psychopathology comes from recent longitudinal research that has found that executive functioning deficits were typically present prior to the development of psychopathology in adolescence, and that deficits in executive functioning predicted change in psychopathological symptoms over the following two years [6]. Given the proposed importance of neurocognitive abilities in psychopathology, there have been vast amounts of research attempting to uncover what particular neurocognitive abilities contribute to each specific diagnosis. This line of research has had little success. Even though neurocognitive deficits are common in most disorders, the nature of neurocognitive deficits within disorders is extensively heterogeneous, see [9]. For example, Martino et al. [10] and Raffard and Bayard [11] found extensively heterogeneous combinations of neurocognitive deficits within their samples of people diagnosed with bipolar disorder and schizophrenia, respectively. Furthermore, even when comparing different disorders, particular neurocognitive deficits cannot differentiate diagnoses [12].

One possible reason for the extensive heterogeneity of the associations between neurocognitive performance and psychopathology is the predominant use of the traditional nosological approach to diagnosis [13]. Traditional nosological approaches to the diagnosis of mental disorder, which use tools such as the DSM, have resulted in high levels of comorbidity and poor diagnostic stability, e.g., [14,15], making the study of any single psychopathological disorder difficult [13]. Further, the overlapping symptoms present between different disorders, as well as the ability for two people to be diagnosed with the same disorder and having no, or very few, common symptoms [16,17], means that finding particular collections of neurocognitive deficits fundamental to any particular disorder is unlikely [9,13,18].

In recent years, to mitigate the issues of comorbidity and diagnostic stability of the traditional nosological approach, there have been calls to move towards dimensional approaches to describing and explaining psychopathology [19,20]. Rather than classifying collections of symptoms into categories known as diagnoses, dimensional approaches typically assess symptoms and organise them into dimensional structures of psychopathology using factor analytic approaches [19,20]. Many such structural models of psychopathology exist. Two structures, the correlated factors model and the bifactor model, have gained the most interest within the literature. The correlated factors model contains a range of symptoms, serving as indicators, and a smaller collection of specific factors (such as internalising, externalising, and thought disorder) that account for the common variance of closely related symptoms. The bifactor model contains the same fundamental components as the correlated factors model but also incorporates a single higher-order general factor (called the *p*-factor) that has been claimed to represent general psychopathology or the propensity toward all psychopathological symptoms [21]. Other common structures include the single-factor model that incorporates the symptom indicators and the *p*-factor, but no specific factors [21].

As structural models of psychopathology do not create diagnostic categories and instead measure dimensionally, it has been suggested that structural models will improve our ability to find more reliable patterns of risk factors and outcomes associated with psychopathology [13,18,20]. While it is important to note that there is a lack of consensus on the substantive interpretation of the factors of psychopathology, in particular the *p*-factor, and their applications to subgroups of a population, for further detail see [18,22,23], structural models of psychopathology offer a useful framework for examining the associations between neurocognitive abilities and psychopathology [13]. Previously, we suggested that it may be possible to find, at a population level, patterns of association between neurocognition and the factors of psychopathology that help explain the differentiation between the factors. That is, factors from structural models such as internalising, externalising, and thought disorder might have discrete patterns of neurocognitive ability associations that differentiate the factors [13]. This finding may provide insight into what neurocognitive abilities are particularly salient for the common variance of collections of different types of symptoms. This knowledge may then inform the starting point for assessment and treatment decisions on the individual level and direct longitudinal work exploring the specific neurocognitive risk factors for collections of psychopathological symptoms. However, there has been a lack of detailed examination of the associations between specific neurocognitive processes and psychopathology factors from different types of structural models. Previous studies of the association between neurocognitive abilities and structural models of psychopathology have typically only reported the bivariate correlations between the factors of psychopathology and neurocognitive tasks and composite scores [21] or used a single neurocognitive ability score [24]. Further, other work has modelled neurocognitive abilities as a factor within the structural models of psychopathology [18,25]. Modelling neurocognition within models of psychopathology, while having several unique strengths [18], does not allow for the exploration of the patterns of association between discreet neurocognitive abilities and the different factors of psychopathology. For example, we called for the use of S-1 bifactor models, with neurocognitive abilities modelled as the general factor, to explore neurocognitive abilities associated with psychopathology [18]. We describe how S-1 bifactor models, with neurocognition modelled as the general factor, offer the unique opportunity of mitigating the issue of the unknown substantive meaning of the *p*-factor. However, we also described how the S-1 bifactor approach is limited as neurocognitive abilities may only be explored as a single factor, and therefore the sole use of this approach means it is not possible to examine the associations of particular neurocognitive abilities with each of the factors of psychopathology [18], an area that is particularly lacking within the literature. Due to this limitation of the S-1 approach, and the complementary information that may be obtained, we suggested that using other modelling approaches (e.g., the correlated factors model) to examine neurocognition in psychopathology remains important. Ultimately, to provide a starting point for assessment and treatment decisions, as well as to inform the future assessment of neurocognitive risk factors of psychopathology, it is important to gain a detailed understanding of the specific relations between various neurocognitive abilities and the different factors of psychopathology found in the literature. Yet, to date, the degree to which psychopathology factors may differ regarding patterns of neurocognitive ability associations is unknown.

In this paper, our first aim was to (1) develop and test the fit of three of the most prominent models of psychopathology within a community sample; (a) the correlated factors model, (b) the bifactor model, and (c) the single factor model, using dimensional symptom measures. Our second aim (2) was to explore the degree to which tasks measuring four prominent neurocognitive components, (a) working memory, (b) shifting, (c) inhibition, and (d) speed of processing, are associated with, and can account for, the factors of psychopathology regarding each model.

## 2. Methods

### 2.1. Participants

Through Prolific, we collected data online from a representative community sample (based on simplified census data on age, gender, and ethnicity) of 425 participants in the USA. Exclusion criteria were (1) any condition or injury that could impact their motor movements, thereby interfering with the participants’ ability to complete the cognitive tasks, and (2) colour blindness or colour perception issues. This study was approved by the Curtin University Human Research Ethics Committee (HRE2021-0105).

### 2.2. Procedure

After providing consent, participants provided demographic information (i.e., age, gender), psychiatric history information (i.e., diagnosis, psychiatric hospital admissions), and information of any psychotropic medication use. Participants then completed measures of substance use (ASSIST V3.1) [26], psychiatric symptoms (the Brief Symptom Inventory) [27], and completed eight neurocognitive tasks. Participants were instructed to complete the survey and tasks in an environment as free from distractions as possible. Online neurocognitive data collection does not allow for controlling the participants’ testing environment or hardware. However, a myriad of research supports the validity and quality of online, crowd-sourced, neurocognitive data and has found participants’ performance comparable to laboratory-based studies [28,29,30,31]. Furthermore, Prolific recently has been shown to obtain behavioural task data practically indistinguishable from in-person lab testing, far outperforming similar crowd-sourcing platforms concerning quality and comparability [32].

### 2.3. Materials

#### 2.3.1. Substance Use and Symptomology

To measure substance use and personal, social, and legal issues related to that use, we used the Alcohol, Smoking, and Substance Involvement Screening Test (ASSIST) V3.1 [26]. The ASSIST assesses the use of tobacco products, alcoholic beverages, cannabis, cocaine, amphetamine-type stimulants, inhalants, sedatives or sleeping pills, hallucinogens, opioids, and other substances. Each participant indicates frequency of use, desire or urge to use, and frequency of health, social, legal, and financial issues related to use for each substance used within the last three months. The ASSIST generates a substance involvement score for each substance assessed. The ASSIST has shown strong reliability and validity in general community samples [26].

Psychiatric symptoms were measured via the Brief Symptom Inventory-53 (BSI) [27]. The BSI is a psychiatric symptom measure that assesses symptoms over the past seven days, and is valid and reliable in both clinical and community samples [33]. The measure is comprised of 53-items, measured on a five-point Likert-type scale, that jointly assess nine symptom dimensions based on the original factor structure. The nine symptom dimensions are somatisation, obsession-compulsion, interpersonal sensitivity, depression, anxiety, hostility, phobic anxiety, paranoid ideation, and psychoticism.

#### 2.3.2. Neurocognitive Abilities

Participants completed eight neurocognitive tasks presented in randomised order. The performance metric for each task that involved both speed of response and accuracy was calculated using the Rate-correct Score (RCS) method [34], in which the number of correct responses is divided by total reaction time (in milliseconds) to provide a metric number of correct responses per millisecond. For the tasks that did not require a speed of response aspect, we used only a metric of accuracy. All tasks were developed in JavaScript. The details of each task are provided below.

##### Working Memory

Working memory was assessed via (1) a digit span task, and (2) a visual working memory task after the visual array task described in Cowan et al. [35]. In the digit span task, participants were presented with number sequences, in which each number remained on screen for 1000 ms. Following the presentation of the last number in each sequence, participants were prompted to enter the previously shown sequence, in order, using an on-screen keypad and their PC mouse. The task was designed so that trials increased in difficulty, starting with a 3-digit sequence and progressing to the most challenging 15-digit sequence. Sequences across trials either increased by one digit for each correct response or decreased in length by one digit for every two consecutive errors made. Participants completed 12 trials of the digit span task, with a maximum digit span possible of 15. The outcome variable used was the maximum digit span across the 12 trials.

In the visual memory task, participants completed 84 trials showing sets of either 4, 6, 8, or 10 coloured dots. The initial presentation of dots remained on-screen for 300 ms, followed by a brief interstimulus interval of 1000 ms before a second set of dots was presented. Participants were instructed to indicate whether a circled dot in the second presentation was different in colour to the initial presentation. Performance was assessed via accuracy on the number of correct responses across all 84 trials.

##### Shifting

Shifting was assessed using a Shape-Number switching task and the Inferring Relevance shifting task [36]. The Shape-Number task was adapted from the Letter-Number task [37]. It consisted of participants completing 96 trials in which, following familiarization blocks, they were required to respond to either the number (i.e., 2 vs. 3 dots) or shape (i.e., square or diamond) as stimuli were presented in a 2 × 2 grid. Stimuli appeared sequentially and in a clockwise pattern, and participants used either the Z or M key on their keyboard to respond. For stimuli that appeared in the top row, participants responded based on their shape. When stimuli appeared on the bottom row, participants responded based on the number of dots. Our outcome variable for the Shape-Number task was the number of correct responses divided by total reaction time. Our outcome variable for the Shape-Number task was the number of correct responses divided by total reaction time.

The Inferring Relevance task [38] was derived from the Wisconsin Card Sorting task [39] and the Intra-Dimensional/Extra-Dimensional Shifts task [40]. This task required that participants use their PC mouse to select one of three different on-screen stimuli, depending on what they believed to be the dimension-to-match, in a given trial. Participants completed 200 trials whereby the dimension-to-match was either ‘shape’ (i.e., squares, triangles, and circles), ‘colour’ (i.e., shapes outlines were either red, green, or yellow), or ‘pattern’ (i.e., within each shape was either grid lines, dots, or waves). The correct dimension-to-match changed after 15–25 consecutive trials of one dimension. As per the WCST, correctly identifying the dimension-to-match occurs initially via trial and error and feedback presentation. However, to increase task difficulty, participants’ certainty of response was interfered with by providing incorrect feedback on 25% of trials [38]. The primary outcome measure was the number of correct responses divided by total reaction time.

##### Inhibition

Inhibition was assessed via computerized versions of (1) the Stroop Task [41] and (2) the Go/NoGo task [42]. The Stroop task comprised a total of 48 trials, with 16 trials each for neutral (four “X”s appeared in one of three colours: blue, red, or green), congruent (words “BLUE”, “RED”, or “GREEN” appeared in colours that matched the meaning of the word presented; i.e., the word “BLUE” appeared in the colour blue), and incongruent (words “BLUE”, “RED”, or “GREEN” appeared in colours that did not match the meaning of the word presented; i.e., the word “RED” appeared in the colour green) conditions. In all trials, participants were required to indicate the colour of letters presented on screen, using their mouse to select one of three corresponding buttons on-screen (“Blue”, “Red”, or “Green”). Participants were asked to select, as quickly as possible, the box that corresponded to the colour of the text presented. Therefore, in the incongruent condition, participants had to inhibit selecting the box that corresponded to the text rather than the colour [41]. The primary outcome variable was the number of correct responses for congruent stimuli divided by the total reaction time for those stimuli, subtracted from the number of correct responses for incongruent stimuli divided by the total reaction time for those stimuli.

The Go/NoGo task [42] used consisted of 120 trials. One of two stimuli, either an “M” or a “W”, was presented on-screen, and participants were instructed to press the space bar as quickly as possible when presented with the “M” (the “Go” stimuli) but not to press the space bar when presented with the “W” (the “NoGo” stimuli). Out of the 120 stimuli, the “Go” stimuli accounted for 80%, while the “NoGo” accounted for 20%. This weighting of “Go and “NoGo” stimuli has been shown to provide adequate variability of errors [38]. Stimuli were presented between 1000 ms and 1550 ms apart, and participants were given 1200 ms to respond. As 80% of the stimuli were “Go” stimuli, when presented with a “NoGo” stimuli, participants were required to actively inhibit the predominant response of pressing the space bar. Our primary outcome variable of the Go/NoGo was the number of correct NoGo omissions divided by the total reaction time of responses.

##### Speed of Processing

Speed of processing was assessed via two tasks, (1) a simple reaction time task and (2) the Inspection Time (IT) task [43]. For the simple reaction time task, participants were instructed to respond to the on-screen presentation of a blue “circle” by pressing the space bar on their keyboard as quickly as possible. Participants completed a total of 40 trials, with each trial separated by an interval of between 1000 ms and 1750 ms (this was to avoid participants preempting responses). The outcome variable for the simple reaction time task was the number of correct responses divided by the total reaction time.

On the IT task, participants were presented with images depicting an alien with two antennae [43]. Four variations of this stimulus were used, showing (1) both short antennae, (2) both long antennae, (3) the left antennae being longer than the right antennae, and (4) the right antennae being longer than the left. The exposure duration of stimuli was manipulated so that stimuli were tested at 4 ms increments, between 6 ms and 62 ms a total of 4 times each, thus comprising a total of 60 trials. After each presentation, a mask was presented on screen, and participants were required to indicate whether the previously shown antennae were the same (via pressing the “Z” key) or were different (via pressing “M”) in length. Our outcome variable was *a + b* where: *a* = the lowest exposure duration for two consecutive blocks where accuracy was at 75% or higher. And *b* = *a* growing sum of exposure duration blocks with greater than 75% accuracy, divided by the number of blocks over 75%. Lower scores, therefore, reflected better performance.

### 2.4. Analysis

The analysis of this data occurred in multiple steps. Step one was to confirm the factor structure of the Brief Symptom Inventory (BSI). We used confirmatory factor analysis (CFA) to test two structures of the BSI from the literature. (1) the original Derogatis and Melisaratos [27] nine-factor/49-item structure, and (2) a more recent six-factor, 40-item structure found by Schwannauer and Chetwynd [44]. Step two of the analysis was used to create the subscale scores for the choice of BSI factor structure, and to examine the bivariate correlations between the demographic, BSI, and ASSIST variables. Step two of the analysis used exploratory factor analysis (EFA), among the BSI subscales and the ASSIST variables, to support the development of the models of psychopathology. Step three consisted of choosing the specific psychopathology factors by devising correlated factors models based on the EFA and conceptual interpretation and using CFA to test the models’ fit. In step four, the four structural models of neurocognition were tested; a correlated factors model, two versions of a bifactor model, and a single-factor model. Finally, step five consisted of assessing partial bivariate correlations (accounting for covariates) between the neurocognition and the factors of psychopathology, and a multivariate multiple regression analysis to examine the degree to which the participants’ performance on the neurocognitive tasks could account for the factors of psychopathology after accounting for covariates and the common variance of the tasks.

For our CFIs, we applied less stringent rules of thumb to indicate a good fitting model, and used these rules in combination with conceptual interpretation when choosing a model from alternatives. This approach was taken due to a smaller sample size with an initial large number of observed variables in [45]: and Greene et al. [46,47] emphasises the use of conceptual interpretation and the minimisation of the reliance on fit measures when choosing models. For our CFAs, an RMSEA of <0.05 indicated a good fit, <0.08 indicated a reasonably good fit, and <0.10 indicated a mediocre fit [48]. An SRMR of <0.09 indicated a good fit [49], while the earlier convention of the TFI and the CFI of =>0.9 was used to indicate a good fit, rather than using the later convention of =>0.95 due to the tendency of a =>0.95 cut off to over reject true-population models with smaller sample sizes (<N = 500; [49]). All factor loadings were required to be significant at the alpha level of <0.05. For all models, the MLR estimator with robust test statistics was used.

Regarding the EFA, an oblique (GeominQ) rotation was used, with an ML estimator, and a model was chosen from alternatives based on information derived from the EFAs, as well as the subsequent CFAs, in combination with theoretical and conceptual interpretation. Therefore, models were chosen based upon an exploratory-confirmatory continuum [47], incorporating the importance of conceptual interpretation of the models.

## 3. Results

After cleaning the data, 25 of the 425 participants were removed due to incomplete data for one or more of the neurocognitive tasks, leaving a final sample of N = 400. The demographic and clinical variables for our final sample can be found in Table 1.

### 3.1. Step-One

First, we confirmed the structure of the BSI by testing the original nine-factor model [27] and the newer six-factor model [44]. The original, nine-factor, BSI structure did not fit the data well, with the CFI and the TLI not meeting the criteria for a good fit (χ^2^ (1091, N = 400) = 2350.07, CFI = 0.867, TLI = 0.857, SRMR = 0.064, RMSEA = 0.066, 90% CI = [0.051, 0.056]), and had multiple non-positive definite identification issues. This suggested that there were multiple redundant items within the factor structure. However, the Schwannauer and Chetwynd [44] six-factor structure provided a “reasonably good fit”, with regards to RMSEA, a “good fit” regarding the SRMR, and bordering on a good fit for the TFI and the CFI (χ^2^ (725, N = 400) = 1518.41, CFI = 0.891, TLI = 0.885, SRMR = 0.058, RMSEA = 0.064, 90% CI = [0.049, 0.055]). The six-factor structure also had no identification issues and very good-to-excellent internal consistency (Cronbach’s Alpha’s ranging from 0.858 to 0.940). Therefore, we concluded that, overall, the six-factor structure provided an adequate fit for the data while offering clearly conceptually interpretable factors. The six-factors, with names devised from examining the contents of each factor, their associated BSI item numbers, the original factor they were placed within the nine-factor solution, and their Cronbach’s Alpha’s, can be found in Table 2. Although the factor named “mental fog” contained only items from the BSI aimed at measuring distress related to obsessive-compulsive symptoms, it was named as such due to the subset of items retained reflecting perceived mental performance in daily life, just one aspect of the obsessive-compulsive phenotype. Example items included “Having to check and double-check what you do” and “Your mind going blank” [27].

### 3.2. Step-Two

Following the choice of the six-factor BSI solution, scores for each of the six factors were created from the relevant BSI items using the original scoring procedure. An “other substances” ASSIST variable was also created by adding together scores from the cocaine, amphetamine, inhalants, sedatives, and hallucinogens categories, as there was little variation within these substances. The combination of less commonly used substances is standard amongst the literature, e.g., [21]. The bivariate associations between the six BSI and the four ASSIST variables were explored. This was done to test for the appropriateness of using each BSI and ASSIST variable in developing our models of psychopathology. The bivariate correlations can be found in Table 3. All BSI and ASSIST variables had significant positive correlations, except for tobacco, which was only significantly associated with one of the six BSI variables (Somatisation). As the development of structural models of psychopathology is grounded in significant positive associations between the variables, tobacco use was not included in the development of the models of psychopathology or any other subsequent analyses.

### 3.3. Step-Three

In step three, we used EFA to inform the development of the specific, second-order factors of psychopathology. Given we had nine observed variables, six BSI variables, and three ASSIST variables, we started by examining a four-factor structure, which is the largest structure with the possibility of at least two observed variables loading onto each factor. The EFAs can be found in table four. For the four-factor EFA, a factor emerged consisting of depression, mental fog, and interpersonal anxiety. This factor also showed a cross-loading between factor two for agoraphobia. Furthermore, a second factor emerged consisting of the cross-loaded agoraphobia variable and somatisation, and a third factor consisting of a single loading > 0.3 in hostility. Finally, a fourth factor emerged consisting of the three substance use variables. Next, we assessed a 3-factor structure. The three-factor structure revealed similar results when compared to the four-factor structure. A factor still emerged consisting of depression, hostility, mental fog, and interpersonal anxiety, but now also included hostility, which was moved from its own factor. Factor two emerged still consisting of somatisation and the agoraphobia cross-loading with factor 1. The third factor contained the three substance use variables. Finally, we tested a two-factor solution. The two-factor solution consisted of a factor accounting for the BSI items and for the ASSIST items. The results of the EFAs are presented in Table 4.

The three and two-factor models provided the most parsimonious solutions, and were chosen to be further explored using CFAs. First, we tested two different three-factor models and two different two-factor models. The first three-factor model tested (a), following the exact structure as the three-factor EFA, and loading agoraphobia onto factor two, due to its slightly stronger loading, and its conceptual relationship to somatisation. The second three-factor CFA tested (b) was the same as the first. However, hostility was loaded onto factor three, with the substance use variables. All of the three factors were allowed to correlate. The first two-factor solution tested (c) was derived directly from the two-factor EFA, but the second two-factor model tested (d), like the three-factor model (b), had hostility loaded on as a factor with the substance use variables. We tested the alternative two and three-factor models for two reasons; the four-factor solution showed hostility loading on a separate factor, not on factor one, and hostility or conduct issues is primarily conceptualized with substance use as an “externalising” factor within the literature, e.g., see [21]. Furthermore, regarding the alternative two-factor solution, by having hostility loading onto a factor with substance use, we tested a model with “Internalising” and “Externalising” factors. These factors have been repeatedly validated and received a great amount of interest throughout the literature [13,21,23,50,51]. For all models, the factors were allowed to correlate.

The two three-factor solutions showed to be a “reasonably good” and “mediocre” fit, respectively. The first model (a) (χ^2^ (24, N = 400) = 34.00, CFI = 0.993, TLI = 0.989, SRMR = 0.020, RMSEA = 0.032, 90% CI = [0.000, 0.052]), with hostility loaded onto factor one provided a marginally better fit than the second model (b) (χ^2^ (24, N = 400) = 73.34, CFI = 0.963, TLI = 0.945, SRMR = 0.057, RMSEA = 0.072, 90% CI = [0.057, 0.087]), with hostility loaded onto factor three with the substance use variables.

Next, we tested the fit of the two variations of the two-factor model. The first two-factor model tested (c), with hostility loading onto factor one, was a “reasonable” fit for the data with regards to the RMSEA, and a good fit for the CFI, TLI, and SRMR (χ^2^ (26, N = 400) = 96.73, CFI = 0.975, TLI = 0.966, SRMR = 0.027, RMSEA = 0.057, 90% CI = [0.042, 0.073]). The alternative two-factor solution tested (d) was a “mediocre”-to-“reasonable” fit for the data with regards to the RMSEA, and a good fit for the CFI, TLI, and the SRMR (χ^2^ (26, N = 400) = 96.73, CFI = 0.942, TLI = 0.926, SRMR = 0.059, RMSEA = 0.081, 90% CI = [0.068, 0.097]).

Given that all of the four CFAs tested provided a fit for the data, each model may have been acceptable to select. However, given that a two-factor “Internalising” and “Externalising” model fitted the data and that there is a large amount of conceptual and empirical evidence supporting the use of these factors, we selected this model as our correlated factors model [13,18,21,23,51].

Next, after developing the choice of the correlated-factors model, we tested the fit of two different bifactor models. Each model tested consisted of the same observed variables and the same specific factors (Internalising and Externalising) as in the correlated factors model, but included a higher-order *p*-factor. Each of the nine observed variables loaded onto the *p*-factor as well as either Internalising or Externalising. What differentiated the models was whether the specific factors were allowed to correlate. In the first model tested (a), the specific factors were not allowed to correlate, but in the second model (b), the specific factors were allowed to correlate. We tested both of these versions of the bifactor model as previous research has applied both types successfully [21,52]

The first bifactor model tested (a), without correlated specific factors fit the data well (χ^2^ (18, N = 400) = 23.82, CFI = 0.996, TLI = 0.992, SRMR = 0.020, RMSEA = 0.029, 90% CI = [0.000, 0.053]). However, none of the three observed variables retained significant loadings on the Internalising specific factor, and hostility did not retain its significant loading on the Externalising factor. Finally, there was also a Heywood case, an observed variable with negative variance (somatisation). These findings are thought to be due to the higher-order *p*-factor subsumed the Internalising specific factor, as well as the variance in hostility accounted for by the Externalising factor. The second bifactor model tested (b), that contained correlated specific factors, also fit the data well (χ^2^ (17, N = 400) = 21.18, CFI = 0.997, TLI = 0.993, SRMR = 0.017, RMSEA = 0.025, 90% CI = [0.000, 0.051]). However, the second model (b) shared many of the same issues as the first (a). For model two (b), none of the observed variables retained significant loadings on Internalising. Hostility also did not retain its significant loading on Externalising. Furthermore, somatisation was also a Heywood case within this model. Overall, for both bifactor models, the *p*-factor subsumes the Internalizing factor. A specific factor being subsumed is relatively common in bifactor models of psychopathology, and previous research has removed the factor subsumed [21]. However, this is now known to be poor practice, as if the subsumed factor is removed, the *p*-factor becomes defined by that removed factor, changing its interpretation, see [18,53]. Therefore, the bifactor model is not appropriate to explore further within this data. The results do, however, suggest a single-factor model may be a good fit for the data.

**Figure 1:**
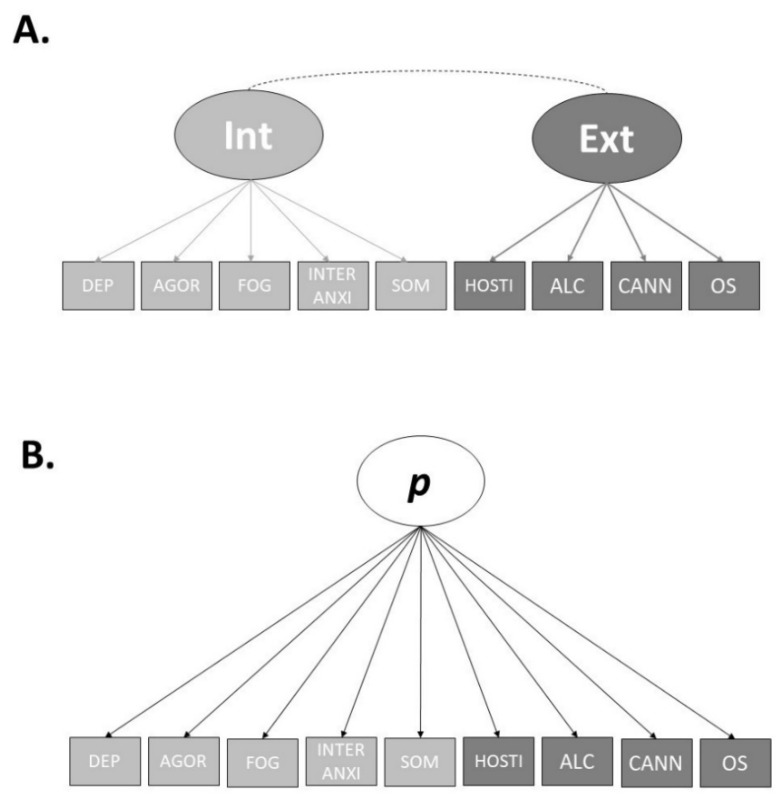
Final Structural Models of Psychopathology. Pictured is the Correlated Factors Model (**A**) and Bifactor Model (**B**). DEP = Depression. AGOR = Agoraphobia, FOG = Mental Fog, INTER ANXI = Interpersonal Anxiety. SOM = Somatisation, HOSTI = Hostility. ALC = Alcohol. CANN = Cannabis. OS = Other Substances.

Lastly, we tested the fit of the single-factor model of psychopathology within our sample. The single-factor model consists of the same nine observed variables used in the other models, however, containing one higher-order *p*-factor and no specific factors. The single factor provided a “mediocre”-to-“reasonably” good fit for with regards to the RMSEA, and a good fit for the CFI, TLI, and SRMR (χ^2^ (27, N = 400) = 98.12, CFI = 0.946, TLI = 0.928, SRMR = 0.062, RMSEA = 0.081, 90% CI = [0.067, 0.095]). All of the nine-observed variables loaded significantly of the *p*-factor. Therefore, we decided to use the (A) correlated factors model and (B) the single-factor model for our examination of the utility of neurocognitive abilities in accounting for the factors of psychopathology. Figure 1 displays two final models.

The factor loadings for both the final correlated factors model and the single-factor model can be found in Table 5. As specified by Caspi et al. [21], we standardised the *p*-factor scores to a mean of 100 and a standard deviation of 15. The internalising and externalising factors were mildly-to-moderately correlated (r = 0.743), while the correlations between the *p*-factor in the single factor model and specific factors in the correlated factors model were strong (*p* and Internalising, r = 0.996; *p* and Externalising, r = 0.799). The *p*-factor and Internalising correlated almost perfectly, indicating the *p*-factor in the single-factor model largely represented Internalising symptoms.

### 3.4. Step-Four

**Figure 2:**
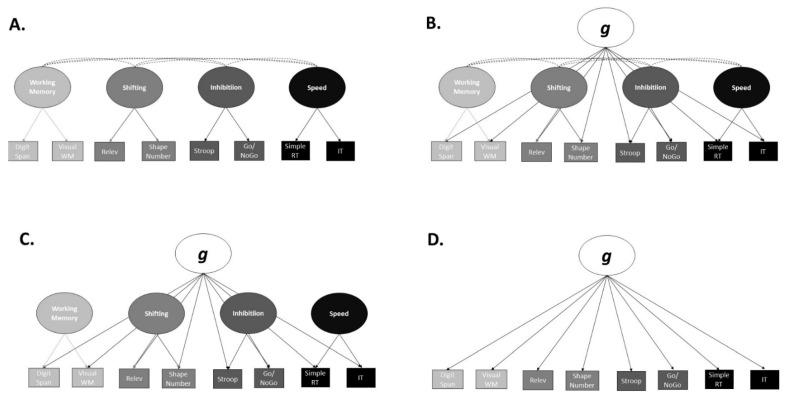
Neurocognitive Structural Models Tested. The Correlated Factors Model (**A**), Correlated Bifactors Model (**B**), Bifactor-Model (**C**), and Single-Factor Model (**D**) Tested. Rele = Inferring Relevance task. WM = Working Memory. RT = Reaction Time. IT = Inspection Time.

After The choice of structural models of psychopathology, we examined the fit of three different structural models of neurocognition; (a) a correlated factors model, (b) a bifactor model with correlated specific factors, (c) a bifactor model without correlated specific factors, and (d) a single factor model. Figure 2 depicts the four models. Unlike our approach to developing the models of psychopathology, we did not precede the confirmatory with exploratory factor analyses. This is because, unlike the components from our measure of psychopathology, we actively chose two specific tasks to measure each theoretically driven neurocognitive component. Therefore, it would be inappropriate to conduct exploratory factor analyses as any alternative structures would forgo the conceptual interpretation and theoretical foundations of the neurocognitive components.

All of the four tested models failed to converge. This may be expected based on the generally low correlations amongst the neurocognitive components. These results suggested it would be most appropriate to examine each neurocognitive test independently within our remaining analyses. Descriptive statistics for the neurocognitive tests are presented in Table 6.

### 3.5. Step-Five

In Step Five we examined the partial (controlling for age and gender) correlations between the neurocognitive tasks and internalising, externalising and the *p*-factor. We controlled for age and gender as both demographic variables were significantly associated with one or more of the factors of psychopathology. Higher age being associated with lower internalising, externalising and *p*-factor scores (internalising, *r* = −0.422, *p* < 0.001; externalising, *r* = −0.348, *p* < 0.001; *p*-factor, *r* = −0.424, *p* < 0.001), and females (males = 1, females = 2) tended to have higher scores on internalising and the *p*-factor each factor (internalising, *r* = 0.201, *p* < 0.001; externalising, *r* = 0.006, *p* = 0.910; *p*-factor, *r* = 0.182, *p* < 0.001). Table 7 shows the bivariate correlations between the neurocognitive tasks and the factors of psychopathology after accounting for age and gender.

Of the eight neurocognitive tasks, after accounting for age and gender, only the two tasks designed to measure the speed of processing were significantly associated with one or more of the factors of psychopathology. Specifically, performance on the simple reaction time task was significantly negatively associated with internalising, externalising, and the *p*-factor. This finding indicates that better performance on the simple reaction time task is significantly associated with lower internalising and externalising symptoms, as well as the *p*-factor score. The Inspection Time task was significantly positively associated with internalising and the *p*-factor, indicating that better performance on the Inspection Time task was associated with lower internalising symptoms and lower *p*-factor scores. Combined, these results indicate that within our data, speed of processing is the primary neurocognitive correlate with higher-order psychopathology.

Next, we used a multivariate multiple regression analysis to examine the degree to which each neurocognitive task could account for unique variance in the psychopathology factors, accounting for age and gender, as well as the common variance amongst the tasks. The model accounted for a significant 23.8% of variance in internalising (*F*(10, 389) = 12.17, *p* < 0.001, R^2^ = 0.238), a significant 15.6% of variance in externalising (F(10, 389) = 8.37, *p* < 0.001, R^2^ = 0.156), and a significant 23.6% of variance in the *p*-factor (F(10, 389) = 12.05, *p* < 0.001, R^2^ = 0.236). Table 8 provides the results of the regression analysis.

Regarding internalising, the simple reaction time task and the Inspection Time task remained significant predictors after accounting for the variance of age and gender, as well as the common variance of the neurocognitive tasks. Simple reaction time performance uniquely accounted for 0.9%, and the inspection time task accounted for 0.8% of the variance in internalising, respectively. This indicates that our tasks assessing the speed of processing are not only significantly associated with internalising after accounting for age and gender but can also account for a significant amount of unique variance in internalising after accounting for age and gender in addition to the common variance from the neurocognitive tasks. However, it is important to acknowledge that combined the unique variance in internalising accounted for by the speed of processing tasks was just 1.7%.

Regarding externalising, simple reaction time performance was a significant predictor of externalising in our model after accounting for age, gender, and the common variance of the remaining neurocognitive tasks. Simple reaction time accounted for a significant 4.0% of unique variance in externalising that could not be explained by age and gender or the common variance of the remaining neurocognitive tasks. However, unlike internalising, our other measure of the speed of processing, the inspection time task, did not account for a significant amount of unique variance in externalising.

Even though the internalising and the *p*-factor were highly correlated, to ensure a full investigation of the study aims and psychopathology factors, it was still important to examine the relations between neurocognitive performance and the general factor. Further, as the internalising and *p*-factor are highly, but not perfectly, correlated, the analyses remained important. Both simple reaction time and inspection time task performance accounted for a significant amount of unique variance in the *p*-factor over and above age, gender, and the common variance of the neurocognitive tasks. Simple reaction time performance accounted for a significant 1.2% of unique variance in the *p*-factor, while performance on the inspection time task accounted for a significant 0.9% of unique variance in the *p*-factor. Overall, our findings suggest that the tasks measuring the speed of processing were the most efficacious when compared to tasks measuring working memory, shifting, and inhibition, in accounting for higher-order psychopathology within our sample.

## 4. Discussion

Strong evidence suggests that deficits in neurocognitive abilities play a role in the aetiology of psychopathology, e.g., [6]. In recent years, evidence has grown for the utility of dimensional structural models of psychopathology as an alternative to traditional nosological diagnostic approaches. However, there is a lack of understanding of how neurocognitive abilities are associated with factors of psychopathology derived from structural models. The aim of this paper was to (1) develop and test the fit of three popular models of psychopathology within a community sample; (a) the correlated factors model, (b) the bifactor model, and (c) the single factor model, using dimensional symptom measures. Our second aim (2) was to explore the degree to which tasks measuring four prominent neurocognitive components, (a) working memory, (b) shifting, (c) inhibition, and (d) speed of processing, are associated with, and can account for, the factors of psychopathology from each model.

Within our sample, only the correlated-factors model and the single factors model fit our data well. The correlated factors model consisted of an internalising factor and an externalising factor. The internalising factor had loadings from depression, agoraphobia, mental fog, interpersonal anxiety, and somatisation, while the externalising factor had loadings from hostility, alcohol use, cannabis use, and other drug use. Our correlated factors model parallels many other correlated factors models found within the literature, e.g., [51]. However, we did not find a third factor, namely “thought disorder”, that is commonly found within the literature. Thought disorder is commonly defined by psychotic symptoms [21], and the absence of thought disorder factors from our models may be explained by the use of the six-factor BSI model over the original nine-factor model. The original nine-factor BSI model, which included a psychoticism factor, did not fit our data well, so the alternative six-factor model, which only included a single psychosis item amongst its factors, was used. Therefore, our six observed variables used to develop the models did not include strong indicators of psychoticism. The bifactor models fit our data well, although they had several non-significant factor loadings and a Heywood case. The good fit of the bifactor models is not surprising given that fit indices bias bifactor models over correlated factors models [46]. However, the single-factor model also fit the data well. The *p*-factor from the single factor model, however, was almost perfectly correlated with the internalising factor from the correlated factors model. This suggests, along with the Heywood cases, that the bifactor models were a poor structure for the data because the *p*-factor primarily represented internalising. The issue of the *p*-factor being malleable and primarily representing a specific factor has been discussed previously in the literature [13,18,23,54]. This represents a limitation of developing an understanding of the substantive meaning of *p.*

We also attempted to fit different structural models of neurocognitive abilities. None of the models fit our data. This was unexpected as previous research generally finds similar models to be a good fit [55]. However, we chose neurocognitive tasks that assessed different aspects of each neurocognitive domain. For example, to measure the speed of processing, we used a simple reaction time task that required participants to respond to a stimulus as quickly and as accurately as possible, as well as an Inspection Time task that did not involve any response speed but instead involved high-speed image processing. Furthermore, to measure shifting, we used a more traditional switching task, the shape-number task, that required participants to switch mental set in response to a known, defined rule (top or bottom of the grid), as well as a less traditional Inferring Relevance switching task [38], that required participants to switch mental set in a probabilistic, more real-world, context. Therefore, given that we measured the breadth of each neurocognitive domain, it is understandable that performance heterogeneity resulted in the models of neurocognition not being a good fit.

We found that, after controlling for age and gender, simple reaction time performance was significantly associated with the internalising and externalising factors concerning the correlated factors model, as well as the *p*-factor regarding the single factors model. We also found additionally, after controlling for age and gender, the IT task performance was significantly associated with internalising and the *p*-factor. However, tasks that measure working memory, shifting, and inhibition were not significantly associated with any of the factors of psychopathology. Furthermore, after accounting for age, gender, and the common variance of the neurocognitive tasks, the simple reaction time task accounted for a significant 0.9%, 4.0%, and 1.2% of the variance in internalising, externalising, and the *p*-factor, respectively. After accounting for age, gender, and the common variance of the neurocognitive tasks, the IT task accounted for a unique 0.8% and 0.9% variance in internalising and the *p*-factor. This suggests that, in our data, tasks that measured speed of processing had the greatest predictive utility, although limited to a combined predictive utility of 1.7%. The lack of predictive utility of working memory, shifting, and inhibition tasks regarding the factors of psychopathology both conflicts and supports findings from the limited research in this area. Caspi et al. [21] found working memory to be significantly associated with internalising and externalising in their correlated factors model as well as to the *p*-factor in their bifactor model. However, paralleling our findings, Caspi et al. [21] also found that a shifting task (i.e., the Trail-Making-Test-B) was not significantly associated with externalising within their correlated factors model but did find it was significantly associated with internalising. Finally, our findings also parallel previous findings [21] in that speed of processing was significantly associated with both internalising and externalising, as well as the *p*-factor. Previously, we suggested that factors from structural models such as internalising, externalising, and the *p*-factor may have discrete patterns of neurocognitive ability associations that differentiate the factors [13]. However, our results suggest that internalising, externalising, and the *p*-factor may not be clearly differentiated by neurocognitive performance, and that processing speed is a common correlate.

The importance of processing speed has been primarily studied in relation to ageing [56,57]. However, there has been growing interest in the role speed processing in psychopathology plays in internalising and externalising disorders and symptoms. For example, a recent systematic review has found that people with major depressive disorder typically have processing speed deficits, and provided evidence that, to compensate for this deficit, people with major depressive disorder are required to use greater cognitive effort to perform daily tasks [58]. Nuño, Gómez-Benito, Carmona, and Pino [58] also suggest that if a task requires a high cognitive demand, deficits in the speed of processing cannot be compensated for by higher cognitive effort, and therefore task performance is poor. Deficits in the speed of processing in depression, therefore, have been suggested to negatively impact occupational performance [58], and this may perpetuate depressive symptoms. Further evidence for the importance of speed of processing in psychopathology is that there is evidence for speed of processing being a reliable cognitive endophenotype for bipolar disorder, with not only people with bipolar disorder experiencing speed of processing deficits, but also significant proportions of relatives of those with bipolar disorder experiencing deficits in speed of processing [59]. Furthermore, it has been found that an intervention designed to train speed of processing in the elderly resulted in a significant reduction in the risk of experiencing depressive symptoms 1 and 5 years post-intervention, while training in perceptual reasoning, or working memory, had no impact [60]. Regarding externalising behaviours, there is a reliable association between alcohol use disorder and speed of information processing deficits [61,62], and speed of processing may not only be a consequence of externalising behaviours but may also be involved in the aetiology of those behaviours. Durazzo et al. [63] found that processing speed deficits significantly predicted relapse in people treated for alcohol dependence after accounting for demographic, psychiatric, metabolic, and clinical covariates. Furthermore, there is evidence that deficits in speed of processing are related to an earlier onset of conduct disorder [64]. Our findings, therefore, parallel previous research proposing the importance of speed of processing within psychopathology. However, our results extend the literature by employing dimensional structures of psychopathology in a representative community sample, showing that deficits in speed of processing are not only related to nosologically defined disorders but also statistically derived dimensions in the general population.

### Limitations of the Research and Directions for Future Research

The data for this study was collected online through Prolific. Therefore, we had little experimental control over the context in which participants completed the survey and tasks and the devices and settings used. However, the range of evidence suggests that the quality of task data collected online through crowd-sourcing platforms, such as prolific, is comparable to in-lab studies [28,29,30,31], and we used the most valid crowd-sourcing platform for this context [32]. Further, we only explored neurocognitive ability’s associations with the factors of psychopathology on the sample and not the individual level. Previously we provided evidence for compensatory neurocognitive profiles on the individual level that can explain the heterogeneous findings between specific neurocognitive abilities and psychopathology [9,13]. Even though at the sample level, measures of working memory, inhibition, and shifting were not significantly associated with the factors of psychopathology, at the individual level, explanatory heterogeneous profiles of neurocognitive performance may exist. For example, there are two individuals with the same high *p*-factor score of 140. Individual One may have a pervasive deficit in working memory while having good shifting, inhibition, and speed of processing ability. However, Individual Two may have a good working memory, shifting, and inhibition ability, but a pervasive deficit in speed of processing. For each individual, their neurocognitive strength and weakness profile may explain their high level of *p*, however, on the sample level (N = 2), no associations would exist between any neurocognitive ability and their level of *p* due to the heterogeneity.

Future research should validate our findings in a laboratory setting to limit potential confounding variables. Future research should also examine the associations between neurocognitive abilities and psychopathology factors on the individual level, exploring potential compensatory neurocognitive profiles and multidimensional explanations.

## 5. Conclusions

In this paper, we explored the associations between neurocognitive abilities and structural models of psychopathology. We found a correlated factors model and single factor model to best fit our psychopathology data. We found tasks measuring speed of processing had the most predictive utility for internalising, externalising, and the *p*-factor. Specifically, poorer performance on the simple reaction time task was significantly associated with higher scores of internalising, externalising, and the *p*-factor, and poorer performance on the Inspection Time Task was significantly associated with higher scores of internalising and the *p*-factor. Tasks that measured working memory, shifting, and inhibition were not significantly associated with psychopathology factors. We found neurocognitive abilities were not differentially associated, but that speed of processing was a common correlate of psychopathology factors.

## Figures and Tables

**Table 1:** Participant Characteristics.

Variable		Mean (SD/%)/Count	Min	Max
Age		44.47 (16.35)	18	83
				
Gender				
	*Male*	194 (48.5%)	-	-
	*Female*	206 (51.5%)	-	-
Diagnosis (Yes/No)				
	*Yes*	114 (28.5%)	-	-
	*No*	286 (71.5%)	-	-
Diagnoses ^a^				
	*Depression*	66 (16.5%)	-	-
	*Generalised Anxiety*	57 (14.2%)	-	-
	*Agoraphobia*	2 (0.5%)	-	-
	*Social Anxiety*	7 (1.8%)	-	-
	*Panic Disorder*	4 (1.0%)	-	-
	*Schizoaffective*	1 (0.3%)	-	-
	*Psychosis*	2 (0.5%)	-	-
	*Eating Disorder*	1 (0.3%)	-	-
	*Cyclothymia*	1 (0.3%)	-	-
	*Bipolar*	17 (4.3%)	-	-
	*OCD*	3 (0.8%)	-	-
	*Impulse Control*	1 (0.3%)	-	-
	*BPD*	3 (0.8%)	-	-
	*PTSD*	19 (4.8%)	-	-
	*Substance Use*	3 (0.8%)	-	-
	*Trichotillomania*	1 (0.3%)	-	-
				
Year of First Diagnosis		2007.69 (10.71)	1980	2021
				
Admitted to a Mental Health Facility (Yes/No)				
	*Yes*	26 (6.5%)	-	-
	*No*	374 (93.5%)	-	
				
Year of First Admission		2003.31 (13.14)	1980	2020
				
Using Psychotropic Medication (Yes/No)				
	*Yes*	60 (15.0%)	-	-
	*No*	340 (85.0%)	-	-

^a^ counts add to over the total sample due to comorbid diagnoses. OCD = Obsessive-Compulsive Disorder. BPD = Borderline Personality Disorder. PTSD = Post-Traumatic Stress Disorder.

**Table 2:** The Six-Factor Model.

Factor Name	Item Numbers	Original Factor	Cronbach’s Alpha
*Depression*			0.940
	17	Depression	
	18	Depression	
	16	Depression	
	14	Psychoticism	
	35	Depression	
	50	Depression	
	44	Anxiety	
*Agoraphobia*			0.865
	8	Phobic Anxiety	
	43	Phobic Anxiety	
	28	Phobic Anxiety	
	31	Phobic Anxiety	
	45	Anxiety	
*Hostility*			0.832
	13	Hostility	
	46	Hostility	
	41	Hostility	
	40	Hostility	
	6	Hostility	
*Mental Fog*			0.909
	36	Obsessive-Compulsive	
	5	Obsessive-Compulsive	
	26	Obsessive-Compulsive	
	32	Obsessive-Compulsive	
	27	Obsessive-Compulsive	
	15	Obsessive-Compulsive	
*Interpersonal Anxiety*			0.904
	21	Interpersonal Sensitivity	
	22	Interpersonal Sensitivity	
	51	Paranoid Ideation	
	20	Interpersonal Sensitivity	
	42	Interpersonal Sensitivity	
	48	Somatisation	
	24	Paranoid Ideation	
	4	Paranoid Ideation	
	10	Paranoid Ideation	
*Somatisation*			0.858
	7	Somatisation	
	30	Somatisation	
	33	Somatisation	
	29	Somatisation	
	23	Somatisation	
	2	Somatisation	
	37	Somatisation	
	1	Anxiety	

**Table 3:** Symptom and Substance Bivariate Correlations.

	Dep	Agor	Host	Fog	Inter. Anx	Somat	Tob	Alc	Cann	Other
**Depression**	1	0.627 **	0.620 **	0.761 **	0.808 **	0.690 **	0.040	0.132 **	0.262 **	0.185 **
**Agoraphobia**	0.627 **	1	0.534 **	0.621 **	0.667 **	0.718 **	0.070	0.133 **	0.244 **	0.211 **
**Hostility**	0.620 **	0.534 **	1	0.668 **	0.698 **	0.655 **	0.022	0.206 **	0.193 **	0.227 **
**Mental Fog**	0.761 **	0.621 **	0.668 **	1	0.765 **	0.745 **	0.070	0.138 **	0.275 **	0.223 **
**Inter. Anxiety**	0.808 **	0.667**	0.698 **	0.765 **	1	0.723 **	0.076	0.150 **	00.257 **	0.233 **
**Somatisation**	0.690 **	0.718 **	0.655 **	0.745 **	0.723 **	1	0.124 *	0.195 **	0.302 **	0.262 **
**Tobacco**	0.040	0.070	0.022	0.070	0.076	0.124 *	1	0.303 **	0.257 **	0.279 **
**Alcohol**	0.132 **	0.133 **	0.206 **	0.138 **	00.150 **	0.195 **	0.303 **	1	0.279 **	0.201 **
**Cannabis**	0.262 **	0.244 **	0.193 **	0.275 **	0.257 **	0.302 **	0.257 **	0.279 **	1	0.349 **
**Other Drugs**	0.185 **	0.211 **	0.227 **	0.223 **	0.233 **	0.262 **	0.279 **	0.201 **	0.349 **	1

*. Correlation is significant at the 0.05 level (two-tailed). **. Correlation is significant at the 0.01 level (two-tailed). Dep = Depression. Agor = Agoraphobia. Host = Hostility. Fog = Mental Fog. Inter. Anx = Interpersonal Anxiety. Somat = Somatisation. Tob = Tobacco. Alc = Alcohol. Cann = Cannabis. Other = Other Substances

**Table 4:** EFA Factor Loadings.

Number of Factors	Item	Factor 1	Factor 2	Factor 3	Factor 4
4					
	Depression	0.936			
	Agoraphobia	0.398	0.418		
	Hostility			0.960	
	Mental Fog	0.617			
	Interpersonal Anxiety	0.797			
	Somatisation		0.975		
	Alcohol				0.376
	Cannabis				0.712
	Other Substances				0.450
3					
	Depression	0.903			-
	Agoraphobia	0.396	0.400		-
	Hostility	0.604			-
	Mental Fog	0.699			-
	Interpersonal Anxiety	0.931			-
	Somatisation		0.981		-
	Alcohol			0.401	-
	Cannabis			0.685	-
	Other Substances			0.489	-
					
2					
	Depression	0.900		-	-
	Agoraphobia	0.711		-	-
	Hostility	0.739		-	-
	Mental Fog	0.862		-	-
	Interpersonal Anxiety	0.920		-	-
	Somatisation	0.772		-	-
	Alcohol		0.429	-	-
	Cannabis		0.595	-	-
	Other Substances		0.524	-	-

Factor loadings < 0.3 are hidden.

**Table 5:** CFA for the Final Two Models.

Model	Factor	Depr	Agor	Fog	Int. Anx.	Soma	Host	Alc	Cann	Other	Int~Ext
Correlated Factors											0.743 **
	*Internalising*	0.862	0.750	0.867	0.896	0.842					
	*Externalising*						0.806	0.227	0.328	0.300	
Single-Factor											
	*p*	0.861	0.750	0.867	0.896	0.842	0.758	0.192	0.316	0.272	

** Correlation is significant at the 0.01 level (two-tailed). Depr = Depression. Agor = Agoraphobia. Fog = Mental fog. Int. Anx. = Interpersonal Anxiety. Soma = Somatisation. Host = Hostility. Alc = Alcohol. Cann = Cannabis Other = Other Substances. Int = Internalising. Ext = Externalising. ~ = correlation.

**Table 6:** Neurocognitive Task Descriptive Statistics.

	Minimum	Maximum	Mean	Std. Deviation
Digit Span	4	14	7.82	1.79
Visual WM	27	74	57.09	8.76
Inferring Relevance	0.0002761	0.0019141	0.0008937	0.0002978
Shape-Number	0.0000799	0.0016144	0.0006979	0.0002565
Stroop	−0.0002305	0.0008982	0.0002402	0.0001444
Go/NoGo	0.0001582	0.0005083	0.0003417	0.0000592
Simple RT	0.0002084	0.0003973	0.0003921	0.0000206
IT	28.67	112.00	67.53	22.08

**Table 7:** Partial Bivariate Correlations Between Neurocognition and Psychopathology.

Control Variables		Digit Span	Vis WM	Infer. Rel.	Shape-Num	Stroop	Go/No Go	Simple RT	IT	Int	Ext	*p*
Age & Gender	Digit Span	1.000	0.045	0.020	0.020	−0.020	0.050	0.060	−0.042	−0.060	0.038	−0.048
	Visual WM	0.045	1.000	0.187 **	0.160 **	0.064	0.122 *	0.203 **	−0.051	−0.053	−0.066	−0.056
	Infer. Rel.	0.020	0.187 **	1.000	0.359 **	0.177 **	0.077	0.014	0.003	−0.016	−0.005	−0.015
	Shape-Number	0.020	0.160 **	0.359 **	1.000	0.100 *	0.107 *	0.078	0.021	−0.016	0.010	−0.013
	Stroop	−0.020	0.064	0.177 **	0.100 *	1.000	0.013	−0.017	−0.044	−0.032	0.031	−0.024
	Go/NoGo	0.050	0.122 *	0.077	0.107 *	0.013	1.000	0.223 **	−0.048	−0.088	−0.061	−0.087
	Simple RT	0.060	0.203 **	0.014	0.078	−0.017	0.223 **	1.000	−0.023	−0.130 **	−0.226 **	−0.148 **
	IT	−0.042	−0.051	0.003	0.021	−0.044	−0.048	−0.023	1.000	0.112 *	0.089	0.113 *
	Internalising	−0.060	−0.053	−0.016	−0.016	−0.032	−0.088	−0.130 **	0.112 *	1.000	0.719 **	0.995 **
	Externalising	0.038	−0.066	−0.005	0.010	0.031	−0.061	−0.226 **	0.089	0.719 **	1.000	0.782 **
	*p*-Factor	−0.048	−0.056	−0.015	−0.013	−0.024	−0.087	−0.148 **	0.113 *	0.995 **	0.782 **	1.000

**. Correlation is significant at the 0.01 level (two-tailed). *. Correlation is significant at the 0.05 level (two-tailed). Vis WM = Visual Working Memory. Infer. Rel. = Inferring Relevance. Shape-Num = Shape-Number RT = Reaction Time. IT = Inspection Time. Int = Internalising. Ext = Externalising. *p* = *p*-factor.

**Table 8:** Multivariate Multiple Regression Analysis.

*Predictors*	Internalising					Externalising					*p*-Factor				
	*B*	β	*p*	*Partial*	*Sr ^2^*	*B*	β	*p*	*Partial*	*Sr ^2^*	*B*	β	*p*	*Partial*	*Sr ^2^*
Age	−0.027	−0.433	<0.001 **	−0.404	0.148	−0.026	−0.346	<0.001 **	−0.321	0.095	−0.398	−0.434	<0.001 **	−0.404	0.149
Gender	0.360	0.174	<0.001 **	0.188	0.028	0.007	0.003	0.951	0.003	<0.001	4.68	0.156	0.001 **	0.170	0.023
Digit Span	−0.024	−0.041	0.360	−0.046	0.002	0.036	0.054	0.249	0.058	0.003	−0.251	−0.030	0.505	−0.034	0.001
Vis WM	−0.002	−0.013	0.783	−0.014	0.001	−0.003	−0.022	0.669	−0.022	<0.001	−0.026	−0.015	0.758	−0.016	<0.001
Infer. Rel.	13.03	0.003	0.950	0.003	<0.001	149.99	0.032	0.555	0.030	0.001	415.57	0.007	0.891	0.006	<0.001
Shape-Num	−9.37	−0.003	0.958	−0.003	<0.001	−61.02	−0.015	0.778	−0.014	<0.001	−214.33	−0.004	0.934	−0.004	<0.001
Stroop	−182.03	−0.025	0.578	−0.028	0.001	264.25	0.031	0.664	0.034	0.001	−1916.14	−0.018	0.686	−0.020	<0.001
Go/NoGo	−840.70	−0.048	0.297	−0.053	0.002	−203.95	−0.010	0.835	−0.011	<0.001	−11229.51	−0.044	0.336	−0.049	0.002
Simple RT	−4973.28	−0.099	0.034 *	−0.107	0.009	−12291.50	−0.209	<0.001 **	−0.214	0.040	−84882.76	−0.117	0.012 *	−0.126	0.012
IT	0.004	0.094	0.040 *	0.104	0.008	0.005	0.082	0.083	0.088	0.006	0.064	0.095	0.037 *	0.105	0.009

*. is significant at the 0.05 level. **. is significant at the 0.01 level. Vis WM = Visual Working Memory. Infer. Rel. = Inferring Relevance. Shape-Num = Shape-Number RT = Reaction Time. IT = Inspection Time.

## Data Availability

Data may be made available by the author upon request, providing requests satisfy our Human Research Ethics Committee approval.
